# Behavioral and Neural Effects of Familiarization on Object-Background Associations

**DOI:** 10.3389/fpsyg.2020.591231

**Published:** 2020-12-07

**Authors:** Oliver Baumann, Jessica McFadyen, Michael S. Humphreys

**Affiliations:** ^1^School of Psychology & Interdisciplinary Centre for the Artificial Mind, Bond University, Gold Coast, QLD, Australia; ^2^Queensland Brain Institute, The University of Queensland, Brisbane, QLD, Australia; ^3^Max Planck UCL Centre for Computational Psychiatry and Ageing Research, London, United Kingdom; ^4^School of Psychology, The University of Queensland, Brisbane, QLD, Australia

**Keywords:** associative memory, functional magnetic resonance imaging, context-dependent, hippocampus, medial temporal lobe

## Abstract

Associative memory is the ability to link together components of stimuli. Previous evidence suggests that prior familiarization with study items affects the nature of the association between stimuli. More specifically, novel stimuli are learned in a more context-dependent fashion than stimuli that have been encountered previously without the current context. In the current study, we first acquired behavioral data from 62 human participants to conceptually replicate this effect. Participants were instructed to memorize multiple object-scene pairs (study phase) and were then tested on their recognition memory for the objects (test phase). Importantly, 1 day prior, participants had been familiarized with half of the object stimuli. During the test phase, the objects were either matched to the same scene as during study (intact pair) or swapped with a different object’s scene (rearranged pair). Our results conceptually replicated the context-dependency effect by showing that breaking up a studied object-context pairing is more detrimental to object recognition performance for non-familiarized objects than for familiarized objects. Second, we used functional magnetic resonance imaging (fMRI) to determine whether medial temporal lobe encoding-related activity patterns are reflective of this familiarity-related context effect. Data acquired from 25 human participants indicated a larger effect of familiarization on encoding-related hippocampal activity for objects presented within a scene context compared to objects presented alone. Our results showed that both retrieval-related accuracy patterns and hippocampal activation patterns were in line with a familiarization-mediated context-dependency effect.

## Introduction

A few brief exposures to a novel stimulus can have an impact on the ability to subsequently learn the familiarized stimulus for a recognition test and to form an “association” between the familiarized stimulus and some other stimulus (Chalmers and Humphreys, 1998, 2003). Furthermore, there are context-dependency effects in which a stimulus is better recognized in the context in which it was learned than in another context (Humphreys, 1976, 1978; Bain and Humphreys, 1988). Such context-dependency effects are indicative of an increased association strength (i.e., more unified encoding) between the context and the to-be-recognized object (Horowitz and Manelis, 1972, 1973; Humphreys and Chalmers, 2016; Humphreys, et al., under review).

A specific observation of interest is that the degree to which items and their context are encoded in unified fashion depends also on their degree of familiarity. Dalton (1993) had participants study face-label pairs, where participants were asked to rate how appropriate the occupation was for that person. Two different study rooms were used, after which half of the studied faces were tested in the original study room and the other half were tested in the other study room. Importantly, 1 week before the study session, half of the faces had been familiarized by being presented four times in a continuous recognition task. The results showed that there was a large decline in performance for participants who were tested in a different room than the original study room for the faces which had not been familiarized, but almost no decline for the faces that had been familiarized. The apparent loss of item information when the global environment association is broken during recognition suggests that the novel face is being learned in a more context-dependent fashion, than the face that has been encountered previously, without the current context. Whether or not there is a similar loss of information when the local face-label pairing is broken up cannot be ascertained in the Dalton (1993) data, since this would have required a condition in which studied face-label pairs are rearranged during testing (Murnane and Phelps, 1993).

The context-dependent memory effect described by Dalton (1993) is intriguing since it is not easily explained by a strengthening effect of prior familiarization but, instead, suggests that prior exposure to an item in a different context also affects the associative binding in subsequent contexts. One avenue to further investigate the effect reported by Dalton is to explore whether encoding of item-context associations is indeed differently affected by item familiarization than the encoding of single items. Behavioral memory experiments, however, do not allow investigation of purely encoding-related effects, since performance can be only measured during the test phase. We therefore used functional magnetic resonance imaging (fMRI) to investigate whether the neural correlates of associative item-context encoding are affected by item familiarization (i.e., whether an item has been encountered without the current context before).

The experimental design by Dalton does not readily lend itself to this purpose. To investigate whether the effect of familiarization differs for single items and item-context pairs, we needed to not only manipulate familiarization but also the context factor (i.e., its presence and absence), preferably using a within-list design. Secondly, investigation of interaction effects (irrespective of whether they are behavioral or neural) requires a high degree of statistical power, which means long study lists will be required to detect such an effect. For the current study, we therefore developed a novel experimental design that caters for these two issues.

In the current study, we first aimed to assess the suitability of our design by conceptually replicating the effects reported by Dalton (1993), such that we expected to observe a decrement in object recognition performance between intact and rearranged test pairs that is significantly greater for non-familiarized objects than for familiarized objects (reflected by a statistical interaction between Object Familiarization and Pair Integrity factors). The initial validation of our design was important since it differed in important aspects from Dalton’s (1993) study. Dalton (1993) observed the context-dependency by manipulating the physical learning environment that was also associated with many objects, whereas our experimental design instantiated context using a large number of scene images that were each uniquely associated with just one object.

Secondly, in the fMRI study, we sought to identify brain regions in which this familiarization-mediated context-dependency effect is expressed, i.e., where familiarization has a bigger effect on encoding-related activity for object-scene pairs compared to objects alone. This would be reflected by a significant statistical interaction between the factors of Item Familiarization (non-familiarized vs. familiarized) and Arrangement (single object vs. pair). We focused our analysis on the medial temporal lobe since it is well-known to play a central role in associative memory (Mayes et al., 2007), and human lesion studies suggest that the hippocampus is involved in single-item and associative memory (Stark et al., 2002). Furthermore, relevant to our current study, a meta-analysis across 74 fMRI studies had shown that the human hippocampus is consistently more strongly activated during encoding of pictorial than verbal material, and is also more strongly activated during associative encoding compared to single item encoding (Kim, 2011).

The behavioral and imaging studies both used a three-part design. Briefly, on Day 1, participants were familiarized with multiple exemplars of several object categories (e.g., cups, buckets, rings, etc., following Baumann, 2018; Baumann et al., 2018). On Day 2, participants were first instructed to memorize multiple object-scene pairs presented to them. For the fMRI study, this was done while participants were in the scanner and included an object-only control condition. Following the encoding phase, participants were tested on how well they could recognize old objects (i.e., objects shown during Encoding) from new objects. The background scenes during recognition were either matched to the same object as in Encoding (“intact”), swapped with a different object’s background (“rearranged”), or were presented with a new object entirely (“new object with old scene”). Half of the objects in Encoding and Recognition were familiarized (i.e., shown in the Familiarization stage) or non-familiarized.

## Materials and Methods

### Experiment 1: Behavioral

#### Participants

We recruited 70 healthy university students studying psychology at The University of Queensland. After removing six participants for incorrectly-timed responses and two for misunderstanding the task, our sample consisted of 62 participants (20 males, 41 females, and 1 unspecified) aged between 17 and 51 years (*M* = 20.16, *SD* = 4.66). Participants were compensated with course credit for their time and each participant provided written consent. This study was approved by the University of Queensland’s Medical Research Ethics Committee.

#### Stimuli

Color photographs of objects were sourced *via* Google image search.^1^ We chose 20 distinct categories of common, similarly-sized (able to be carried) objects: backpack, book, bowl, bowtie, bucket, butterfly, clock, cupcake, hat, kettle, key, lamp, mouse, mug, pen, ring, sunglasses, telephone, toothbrush, and umbrella (we located 16 exemplars of each category). Background scenes were also sourced *via* a Google image search, where we selected 200 scenes of unique interior and exterior locations, such as a baseball field, the deck of a ship, an elevator, a gym, a laundromat, a garden, etc.

**Figure 1:**
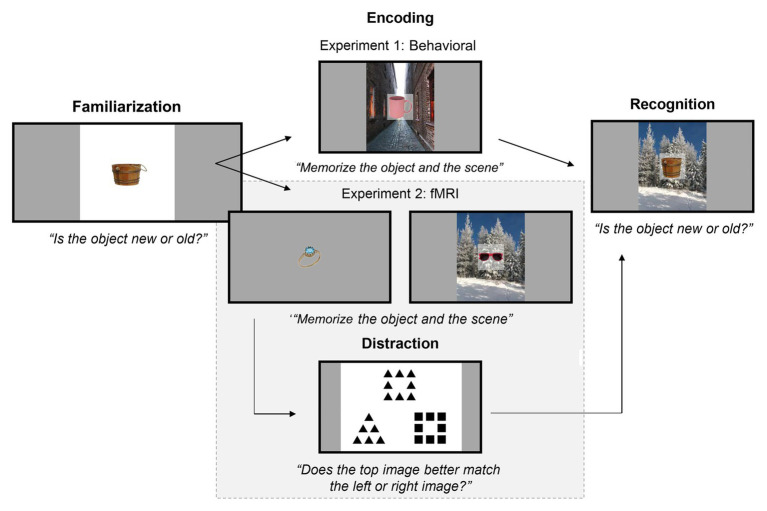
Experimental paradigm for Familiarization on Day 1 and Encoding and Recognition on Day 2, for both experiments. On Day 1, participants were familiarized with exemplars from 10 object categories. On Day 2, participants memorized object-scene pairs (Experiments 1 and 2) and objects alone (Experiment 2). Afterwards, participants were tested on how well they could recognize old objects (i.e., objects shown during Encoding) from new objects. The background scenes during recognition were either matched to the same object as in Encoding (“intact”), swapped with a different object’s background (“rearranged”), or were with a new object entirely (“new object with old scene”). Half of the objects in Encoding and Recognition were familiarized (i.e., shown in the Familiarization stage) or non-familiarized. Note that, in Experiment 2 (highlighted by the gray box), there was an additional distraction task in between Encoding and Recognition, where participants subjectively matched images that shared either global or local geometric properties.

Stimuli were presented at 1920 × 1080 resolution on a 22” LCD monitor, at a 100-cm viewing distance. Objects were presented at a maximum of 300 × 300 pixels, i.e., 4.24 × 4.24° visual angle (objects naturally varied in their aspect ratios, see Figure 1) and scenes always had a size of 800 × 1,000 pixels, i.e., 11.27 × 14.06° visual angle. For the Familiarization stage on Day 1, objects were presented against a white background. For the Study and Test stages on Day 2, objects were presented over the background scenes (see Figure 1).

#### Procedure

The experiment was run through PsychToolbox v3.0.14 (Kleiner et al., 2007) in MATLAB R2016a (The MathWorks, MA, United States). On Day 1, participants were instructed to memorize each object presented to them and to report whether each object was new (“n” key) if it was the first time it had been presented or old (“o” key) if it was the second time it had been presented. On each trial, an initial fixation cross was shown for an inter-stimulus interval of 0.5 s. The object was then shown on a white background for 5 s, during which participants had to make their response. Responses slower than 5 s were not recorded. Stimuli remained on-screen for the entire duration. A total of 250 objects were presented, consisting of 10 object categories with 12 exemplars each shown twice, plus an additional exemplar from each category shown toward the end, so that not all responses at the end would be “old” responses.

On Day 2, participants were told they would complete a memory task unrelated to the task from Day 1. For the initial Encoding stage, participants were instructed to memorize each object-scene pair presented to them, and to specifically remember which object was presented with which background scene (pilot testing had revealed to us that implicit encoding leads to very low memory accuracy for associations). Participants were not required to make responses. Stimuli were presented for 5 s each, preceded by a fixation cross for 0.5 s. Ninety objects from the Familiarization stage were shown again, plus 90 non-familiarized objects (nine exemplars from 10 additional categories – hence, 10 categories were familiar and 10 were unfamiliar), and each object was presented with a unique background scene.

After completing the Encoding stage, participants then immediately completed the Recognition stage, in which they were tested on how well they could recognize old objects (i.e., objects shown during Encoding) from new objects. They were not required to judge the novelty of the background scenes. In total, 180 objects with a background scene were presented and there were 30 trials per familiarity condition (familiarized and non-familiarized) and per pairing condition (intact, rearranged, and new object with old scene). Note that the assignment of exemplar stimuli (i.e., which of the 16 exemplar images were chosen per category) was completely randomized for each participant.

### Experiment 2: fMRI

#### Participants

We recruited 25 healthy university students (8 males and 17 females) aged between 19 and 52 years (*M* = 23.72, *SD* = 7.00) studying psychology at the University of Queensland. Due to a technical error recognition performance in the object alone conditions was not recorded in three participants. Those conditions, however, only served as a control for the imaging analysis and behavioral performance. Participants were compensated with course credit for their time and each participant provided written consent. This study was approved by the University of Queensland’s Medical Research Ethics Committee.

#### Stimuli and Procedure

The stimuli^1^ and procedure were similar to those in Experiment 1, but some modifications were made to accommodate the fMRI component. On Day 1, participants completed the same Familiarization procedure as in Experiment 1 except that participants were presented with a total of 330 objects instead of 250, consisting of 10 object categories with 16 exemplars instead of 12 each shown twice, plus an additional exemplar from each category shown toward the end so that not all responses at the end would be “old” responses.

On Day 2, participants completed the Encoding stage inside the MRI scanner and then the Recognition stage on a computer outside of the MRI scanner room. Stimuli were presented using a liquid crystal display projector (60-Hz refresh rate, resolution 1,920 × 1,080 pixels). The distance from the eyes to the screen (*via* a mirror) was approximately 100 cm. In the Encoding stage, participants were presented with 240 objects for 3 s each, consisting of the 120 familiarized objects from the day prior plus 120 new non-familiarized objects (for each condition 12 exemplars from 10 distinct categories). Within each familiar and unfamiliar category, half of the objects were presented on their own, while the other half was each presented with a unique background scene. This was done so that we could contrast the BOLD signal evoked by object familiarity alone with that evoked by the object-scene pairs. Participants were instructed to memorize the objects presented on their own and to also memorize the unified object-scene pairs that were presented. The order of stimulus presentation was optimized for fMRI using Chris Rorden’s fMRI Design Simulator.^2^ The design parameters were set for an event-related random ISI (minimum = 1 s, maximum = 1.5 s). After exiting the scanner, participants completed a short (approximately 3 min) global-local similarity judgment task (Harrison and Stiles, 2009) to help reduce any ceiling effects in the subsequent Recognition stage.

The Recognition stage was the same as in Experiment 1 except that 200 stimuli were presented, consisting of 80 objects without a background scene (half familiarized/non-familiarized and half new/old) and 120 objects with a background scene (half familiarized/non-familiarized; intact, rearranged, or new object with old scene). Thus, there were 20 trials in each of these 10 conditions (familiarized or non-familiarized × intact, rearranged, new object with old scene, old object alone, or new object alone). Like in the behavioral study, participants were tested on how well they could recognize old objects (i.e., objects shown during Encoding) from new objects and were not required to judge the novelty of the background scenes.

#### MRI Acquisition

We acquired structural and functional brain images using a 3 T Siemens Prisma MRI scanner (Erlangen, Germany) and a 32-channel head coil. Participants lay in a supine position and viewed stimuli *via* a rear-projection mirror mounted on the head coil. Anatomical T1-weighted images were acquired first using an MP2RAGE sequence (*TR* = 4 s, *TE* = 2.99 ms, *FA* = 6°, *FOV* = 140 mm × 256 mm × 154 mm, resolution = 0.8 mm isotropic). Functional T2*-weighted images were acquired using a GRE EPI sequence (*TR* = 1.8 s, *TE* = 30 ms, *FA* = 80°, *FOV* = 192 mm × 192 mm × 96 mm, matrix = 64 × 64, in-plane resolution = 3 mm isotropic). We acquired 29 slices with a width of 3 mm and a 10% inter-slice gap. A total of 290 functional volumes were acquired and we discarded the first eight volumes. Stimulus-onset was synchronized with volume acquisition at delays of 0, 0.9, 1.8, and 2.7 s so that we sampled points across the BOLD response. The image window was positioned so that the hippocampus was included, thus cropping out dorsal brain areas. A gradient echo field map was also acquired (*TR* = 0.4 s, TE 1 = 4.92 ms, TE 2–7.38 ms, *FA* = 60°, *FOV* = 192 mm × 192 mm × 135 mm, 36 slices, resolution = 3 mm isotropic, 3 mm slice thickness).

#### MRI Pre-processing and Analysis

MRI data were preprocessed and analyzed in SPM12 (Wellcome Trust Centre for Neuroimaging, London, United Kingdom) implemented in MATLAB R2016a (The MathWorks, Inc., MA, United States). We followed the preprocessing steps outlined in the SPM12 manual (Ashburner et al., 2014) but using the highest quality and highest degree interpolation options. First, we conducted slice time correction using the first slice as a reference. We then created a voxel displacement map with the FieldMap Toolbox v2.0 using magnitude and phase images from the gradient echo field map sequence. This map was used in the realignment stage to unwarp any field map inhomogeneities. We then co-registered each participant’s structural T1 image and normalized the functional images to MNI space. Finally, we applied Linear Model of Global Signal detrending (Macey et al., 2004) to the functional images and spatially smoothed them using an 8 mm Gaussian FWHM kernel.

We entered these pre-processed images into a first-level analysis per participant. We modeled each of the four conditions (familiarized object-scene, non-familiarized object-scene, familiarized object-alone, and non-familiarized object-alone) along with six movement regressors estimated during slice realignment (three translations and three rotations) as a boxcar function convolved with the canonical hemodynamic response function. The statistical parametric map was masked using each participant’s brain-extracted anatomical image in standard space. Specific one-sample t-contrasts were then calculated for the four simple main effects of our 2 × 2 factor design namely, encoding related-activity for (1) non-familiarized object-scene pairs, (2) familiarized object-scene pairs, (3) non-familiarized objects alone, and (4) familiarized objects alone. On the second level, we used the resulting set of contrast images to perform a factorial interaction analysis across participants.

## Results

### Experiment 1: Behavioral

**Figure 2:**
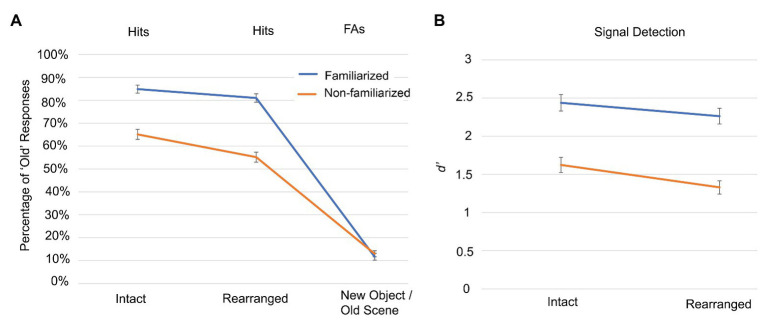
Experiment 1 object recognition accuracy for different pairings with background scenes. **(A)** The hit rates and false alarms (FAs) are shown for participants responding to whether objects presented in the test phase had been presented in the previous study phase. Objects could be familiarized (blue) and non-familiarized (orange), and could be presented in intact, rearranged, and new-object/old-scene pairings. **(B)** Signal detection performance is indicated by D prime (d’) scores, using the corresponding new object condition, to determine the false alarm rate. Error bars indicate standard error of the mean.

We conducted a two (familiarized and non-familiarized) by two (intact and rearranged) repeated-measures ANOVA on the signal detection data to examine how familiarization and the object-scene pairing might interactively influence object recognition. To compute d’, participant data were corrected by adding 1/n trials to each response frequency – the so-called log-linear rule (Hautus and Lee, 1998) – to avoid distortion due to confidence rating frequencies of zero. This method is superior to other solutions for problematic data that include deletion or substitution since it results in less biased estimates of sensitivity (Hautus, 1995). We observed significant main effects of pairing and familiarization, as well as their interaction. Signal detection was significantly better for objects presented in intact (*M* = 2.030, *SEM* = 0.096) than rearranged (*M* = 1.796, *SEM* = 0.086) pairs [*F*(1,61) = 32.439, *p* < 0.001, *η_p_*^2^ = 0.347], and was also better for familiarized (*M* = 2.350, *SEM* = 0.102) than non-familiarized (*M* = 1.476, *SEM* = 0.090) objects [*F*(1,61) = 140.519, *p* < 0.001, *η_p_*^2^ = 0.697]. Critically, we observed a significant interaction [*F*(1,61) = 4.077, *p* = 0.048, *η_p_*^2^ = 0.063], such that signal detection was even more impaired by non-familiarization when the pairs were rearranged (see Figure 2). Thus Experiment 1 successfully replicated Dalton’s (1993) findings that familiarization improved object recognition and reduced the effect of testing in a context different than the study context.

### Experiment 2: fMRI

#### Recognition Performance

Overall, object recognition was best for objects alone (*M* = 1.761, *SEM* = 0.131) than intact (*M* = 1.523, *SEM* = 0.128; *t*(21) = 2.929, *p* = 0.008), which was better than rearranged pairs (*M* = 1.312, *SEM* = 0.118; *t*(21) = 2.635, *p* = 0.015). Signal detection was also better overall for familiarized (*M* = 1.936, *SEM* = 0.149) than non-familiarized (*M* = 1.127, *SEM* = 0.105) objects [*F*(1,24) = 54.759, *p* = 2.808 × 10^−7^, *η_p_*^2^ = 0.723]. The greater overall recognition of familiarized objects was in line with those found in Experiment 1 and confirms that participant successfully engaged with the task. Unlike Experiment 1, the interaction effect in Experiment 2 was not significant (*F*(2,24) = 2.062, *p* = 0.164, *η_p_*^2^ = 0.079). It is important to note that the aim of Experiment 2 was to investigate the encoding-related neural correlates and the sample size was unlikely to provide enough power to reliably detect the interaction effect.

#### fMRI Analysis

**Figure 3:**
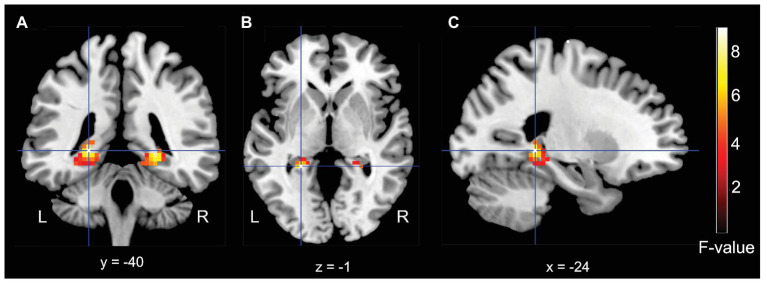
Results for the medial-temporal volume of interest analysis. **(A)** Coronal, **(B)** Axial, and **(C)** Sagittal (left) MRI brain slices depicting a bilateral posterior hippocampal regions that showed a significant interaction effect for the factors of Familiarization (non-familiarized vs. familiarized) and Arrangement (single object vs. pair).

Based on earlier fMRI studies in the area of associative encoding-related activity in the medial temporal lobe (e.g., Hayes et al., 2007; Haskins et al., 2008), we were expecting small effect sizes. We therefore applied a theoretically-guided small-volume correction with a mask that combined the left and right hippocampus and parahippocampal gyrus, using the automated anatomical labeling (AAL) atlas (Tzourio-Mazoyer et al., 2002) and WFU PickAtlas tool (Maldjian et al., 2003). Further, in line with studies by Hayes et al. (2007) and Haskins et al. (2008), we employed a cluster analysis (voxel threshold *p* = 0.05, uncorrected; cluster-defining threshold 25 contiguous voxels). Using this approach we observed a left (peak voxel = −24, −40, −1, cluster size = 47; *F* = 8.96) and a right (peak voxel = 24, −40, −4; cluster size = 43; *F* = 7.90; see Figure 3) posterior hippocampal activity cluster that exhibit a significant interaction effect for the factors Item Familiarization (non-familiarized vs. familiarized) and Arrangement (single object vs. pair).

Finally, we conducted an exploratory whole-brain analysis (voxel threshold *p* = 0.001, uncorrected; cluster-defining threshold 25 contiguous voxels), to identify brain regions that might also show an interaction effect, which did not reveal any additional activations.

**Figure 4:**
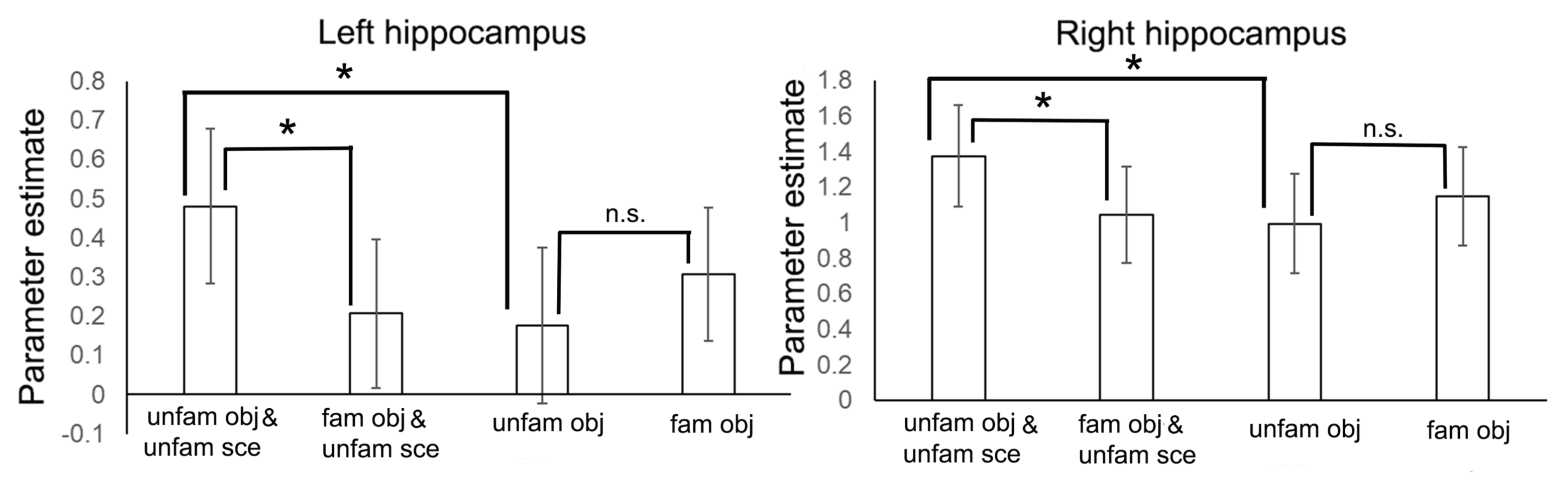
Parameter estimates (±1 SE) from the interaction analysis shown separately for the four conditions and for left and right hippocampus. *p < 0.05.

In a second step, to identify which condition was driving the interaction effect in the hippocampus, we extracted the parameter estimates from the two activation maxima yielded by the factorial analysis separately for our four conditions. As can be seen in Figure 4, the interaction was driven by significantly stronger activity in the non-familiarized object-scene condition relative to the familiarized object-scene condition (two-tailed dependent *t*-test, left hippocampus: *t* = 2.30, *p* = 0.030; right hippocampus: *t* = 2.25, *p* = 0.034), and more similar levels of activity in the two object alone conditions (two-tailed dependent *t*-test, left hippocampus: *t* = 1.60, *p* = 0.124; right hippocampus: *t* = 1.59, *p* = 0.124).

## Discussion

First, our behavioral experiment conceptually replicated the context-dependency effect (Dalton, 1993) by showing that the decrement in object recognition performance between intact and rearranged test pairs was significantly greater for non-familiarized objects than for familiarized objects (as established by a significant interaction between the factors of object familiarization and pair integrity).

Second, our fMRI results demonstrated a context-dependency effect mediated by familiarization, such that there was a larger effect of familiarization on hippocampal activity during encoding for objects presented within a scene context compared to objects presented alone. This was reflected by significantly stronger activity in the non-familiarized object-scene condition relative to the familiarized object-scene condition, whereas activity was more similar between the two object-alone conditions.

The relationship between hippocampal activity and task and performance factor is, however, complex. A recent meta-analysis has shown that hippocampal encoding-related activity is indicative of both a negative relation with item familiarity (also called repetition suppression effect) and a positive relation with successful subsequent memory performance (Kim, 2019). The anatomical overlap between those two processes, which are both related to greater memory performance, suggest that hippocampal activity (at least at the level of resolution afforded by fMRI) cannot serve as a straightforward indicator of memory strength.

Our interpretation, that the increased activity for non-familiarized object-scenes pairs was caused by a greater context-dependency, receives indirect support from a series of fMRI experiments that suggest hippocampal activity is primarily driven by the amount of contextual information being encoded during a study phase or retrieved during a test period (Rugg et al., 2012). Specifically, these experiments reported that hippocampal encoding activity was directly related to the amount of contextual detail (i.e., source information) participants could recount in the test phase. This was complemented by the observation that hippocampal activity during retrieval increased with the amount of information encoded during study (i.e., 1 s vs. 6 s study period). Finally, further support for the idea that the hippocampal activity in our study is indeed related to a context-dependency effect comes from an fMRI study that also observed a relationship between object-background integration and greater hippocampal activity (Memel and Ryan, 2017). In this study, visual integration was varied by presenting an object either next to a scene or visually integrated within a scene. Visual integration not only improved associative memory accuracy but also resulted in increased hippocampal activation during the encoding of visually-integrated object-scene pairs.

Based on behavioral observations and modeling, Popov and Reder (2020) proposed that encoding items with context consumes more resources than encoding single items, since it requires the encoding of item and context, as well as binding the two items together. Additionally, they propose that these operations consume more resources when stimulus representations are weaker (i.e., less familiar). These effects are assumed to be cumulative and the pool of resources to be limited. Therefore, an alternative interpretation of the behavioral interaction effect reported in our study (and that of Dalton, 1993) is that the demands of associative encoding of non-familiarized stimuli exceeded processing demands, and therefore led to a non-linear decrease in recognition accuracy. In other words, according to Popov and Reder, memory strength should be weakest for unfamiliar items that are subjected to associative encoding. The application of this interpretation to our imaging results is, however, more challenging since, as mentioned earlier, encoding-related hippocampal activity has been shown to be negatively related to familiarization (Kim, 2019) while also positively relating to recognition accuracy (Song et al., 2011). Therefore, taken together, our behavioral and imaging results are better explained by a context-dependency effect. The mechanisms of associative encoding are a complex problem that require further investigations.

## Data Availability Statement

The datasets presented in this study can be found in online repositories. The names of the repository/repositories and accession number(s) can be found at: Open Science Framework: https://osf.io/kf2sp/.

## Ethics Statement

The studies involving human participants were reviewed and approved by Human Research Ethics Committee of The University of Queensland. The patients/participants provided their written informed consent to participate in this study.

## Author Contributions

OB, JM, and MH contributed to conception, design, and manuscript writing. OB and JM contributed to data analysis. All authors contributed to manuscript revision and have read and approved the submitted version.

### Conflict of Interest

The authors declare that the research was conducted in the absence of any commercial or financial relationships that could be construed as a potential conflict of interest.

## Footnotes

^1^The stimulus material will be made available upon request.

^2^https://www.mccauslandcenter.sc.edu/crnl/tools/fmristim

## References

[ref1] Ashburner J. Barnes G. Chen C.-C. Daunizeau J. Flandin G. Friston K. (2014). SPM12 manual. London, UK: Wellcome Trust Centre for neuroimaging.

[ref2] Bain J. D. Humphreys M. S. (1988). “The relational context effect: cues, meanings or configurations?” in Memory in context: Context in memory. eds. Davies G. Thomson D. (London: Wiley), 97–138.

[ref3] Baumann O. (2018). Auditory-induced negative emotions increase recognition accuracy for visual scenes under conditions of high visual interference. Front. Psychol. 9:2374. 10.3389/fpsyg.2018.02374, PMID: 30542313 PMC6277761

[ref4] Baumann O. Vromen J. M. G. Boddy A. C. Crawshaw E. Humphreys M. S. (2018). Category-length and category-strength effects using images of scenes. Mem. Cogn. 46, 234–1247. 10.3758/s13421-018-0833-5 29931621

[ref5] Chalmers K. A. Humphreys M. S. (1998). Role of generalized and episode specific memories in the word frequency effect in recognition. J. Exp. Psychol. Learn. Mem. Cogn. 24, 610–632. 10.1037/0278-7393.24.3.610

[ref6] Chalmers K. A. Humphreys M. S. (2003). Experimental manipulation of prior experience: effects on item and associative recognition. Memory 11, 233–246. 10.1080/09658210244000009, PMID: 12908673

[ref7] Dalton P. (1993). The role of stimulus familiarity in context-dependent recognition. Mem. Cogn. 21, 223–234. 10.3758/BF03202735 8469131

[ref8] Harrison T. B. Stiles J. (2009). Hierarchical forms processing in adults and children. J. Exp. Child Psychol. 103, 222–240. 10.1016/j.jecp.2008.09.004, PMID: 18973909

[ref9] Haskins A. L. Yonelinas A. P. Quamme J. R. Ranganath C. (2008). Perirhinal cortex supports encoding and familiarity-based recognition of novel associations. Neuron 59, 554–560. 10.1016/j.neuron.2008.07.035, PMID: 18760692

[ref10] Hautus M. J. (1995). Corrections for extreme proportions and their biasing effects on estimated values of d′. Behav. Res. Methods Instrum. Comput. 27, 46–51. 10.3758/BF03203619

[ref11] Hautus M. J. Lee A. J. (1998). The dispersions of estimates of sensitivity obtained from four psychophysical procedures: implications for experimental design. Percept. Psychophys. 60, 638–649. 10.3758/BF03206051, PMID: 9628995

[ref12] Hayes S. M. Nadel L. Ryan L. (2007). The effect of scene context on episodic object recognition: parahippocampal cortex mediates memory encoding and retrieval success. Hippocampus 17, 873–889. 10.1002/hipo.20319, PMID: 17604348 PMC3615418

[ref13] Horowitz L. M. Manelis L. (1972). “Towards a theory of redintegrative memory” in The psychology of learning and motivation. Vol. 5 eds. Bower G. H. Spence J. T. (New York: Academic Press).

[ref14] Horowitz L. M. Manelis L. (1973). Recognition and cued recall of idioms and phrases. J. Exp. Psychol. 100, 291–296. 10.1037/h0035468

[ref15] Humphreys M. S. (1976). Relational information and the context effect in recognition memory. Mem. Cogn. 4, 221–232. 10.3758/BF03213167 21287026

[ref16] Humphreys M. S. (1978). Item and relational information: a case for context independent retrieval. J. Verbal Learn. Verbal Behav. 17, 179–187. 10.1016/S0022-5371(78)90137-8

[ref17] Humphreys M. S. Chalmers K. A. (2016). Thinking about human memory. Cambridge: Cambridge University Press.

[ref18] Kim H. (2011). Neural activity that predicts subsequent memory and forgetting: a meta-analysis of 74 fMRI studies. NeuroImage 54, 2446–2461. 10.1016/j.neuroimage.2010.09.045, PMID: 20869446

[ref19] Kim H. (2019). Neural correlates of explicit and implicit memory at encoding and retrieval: a unified framework and meta-analysis of functional neuroimaging studies. Biol. Psychol. 145, 96–111. 10.1016/j.biopsycho.2019.04.006, PMID: 31034858

[ref20] Kleiner M. Brainard D. Pelli D. (2007). What’s new in Psychtoolbox-3. Perception 36, 1–16.

[ref21] Macey P. M. Macey K. E. Kumar R. Harper R. M. (2004). A method for removal of global effects from fMRI time series. NeuroImage 22, 360–366. 10.1016/j.neuroimage.2003.12.042, PMID: 15110027

[ref22] Maldjian J. A. Laurienti P. J. Kraft R. A. Burdettea J. H. (2003). An automated method for neuroanatomic and cytoarchitectonic atlas-based interrogation of fMRI data sets. NeuroImage 19, 1233–1239. 10.1016/S1053-8119(03)00169-1, PMID: 12880848

[ref23] Mayes A. Montaldi D. Migo E. (2007). Associative memory and the medial temporal lobes. Trends Cogn. Sci. 11, 126–135. 10.1016/j.tics.2006.12.003, PMID: 17270487

[ref24] Memel M. Ryan L. (2017). Visual integration enhances associative memory equally for young and older adults without reducing hippocampal encoding activation. Neuropsychologia 100, 195–206. 10.1016/j.neuropsychologia.2017.04.031, PMID: 28456521

[ref25] Murnane K. Phelps M. P. (1993). A global activation approach to changes in environmental context on recognition. J. Exp. Psychol. Learn. Mem. Cogn. 19, 882–894. 10.1037/0278-7393.19.4.882

[ref27] Popov V. Reder L. M. (2020). Frequency effects on memory: a resource-limited theory. Psychol. Rev. 127, 1–46. 10.1037/rev0000161, PMID: 31524424

[ref28] Rugg M. D. Vilberg K. L. Mattson J. T. Yu S. S. Johnson J. D. Suzuki M. (2012). Item memory, context memory and the hippocampus: fMRI evidence. Neuropsychologia 50, 3070–3079. 10.1016/j.neuropsychologia.2012.06.004, PMID: 22732490 PMC3472091

[ref500] Song Z. Jeneson A. Squire L. R. (2011). Medial temporal lobe function and recognition memory: a novel approach to separating the contribution of recollection and familiarity. J. Neurosci. 31, 16026–16032. 10.1523/JNEUROSCI.3012-11.2011 22049444 PMC3227550

[ref29] Stark C. E. Bayley P. J. Squire L. R. (2002). Recognition memory for single items and for associations is similarly impaired following damage to the hippocampal region. Learn. Mem. 9, 238–242. 10.1101/lm.51802, PMID: 12359833 PMC187132

[ref30] Tzourio-Mazoyer N. Landeau B. Papathanassiou D. Crivello F. Etard O. Delcroix N.. (2002). Automated anatomical labeling of activations in SPM using a macroscopic anatomical parcellation of the MNI MRI single-subject brain. NeuroImage 15, 273–289. 10.1006/nimg.2001.0978, PMID: 11771995

